# Discovery and Evaluation of Protein Biomarkers as a Signature of Wellness in Late-Stage Cancer Patients in Early Phase Clinical Trials

**DOI:** 10.3390/cancers13102443

**Published:** 2021-05-18

**Authors:** Bethany Geary, Erin Peat, Sarah Dransfield, Natalie Cook, Fiona Thistlethwaite, Donna Graham, Louise Carter, Andrew Hughes, Matthew G. Krebs, Anthony D. Whetton

**Affiliations:** 1 Stoller Biomarker Discovery Centre, Faculty of Biology, Medicine and Health, University of Manchester, Manchester M13 9NQ, UK; bethany.geary@manchester.ac.uk; 2 Division of Cancer Sciences, Faculty of Biology, Medicine and Health, University of Manchester, Manchester M13 9PL, UK; fiona.thistlethwaite@nhs.net (F.T.); louise.carter24@nhs.net (L.C.); Andrew.hughes@manchester.ac.uk (A.H.); 3 The Christie NHS Foundation Trust, Manchester Academic Health Science Centre, Manchester M20 4BX, UK; erin.peat@postgrad.manchester.ac.uk (E.P.); sarah.dransfield@christie.nhs.uk (S.D.); natalie.cook17@nhs.net (N.C.); donna.graham8@nhs.net (D.G.); 4 Manchester National Institute for Health Research Biomedical Research Centre, Manchester M13 9WL, UK

**Keywords:** biomarkers, cancer, swath, proteomics

## Abstract

**Simple Summary:**

TARGET (tumour characterisation to guide experimental targeted therapy) matches patients entering early phase cancer clinical trials to the best treatment for them based on their genetics. Selecting only fit patients for these trials means less patients need to be recruited. Fit patients have a life expectancy of >three months. Performance status (PS) is used to measure life expectancy and is decided by doctors asking questions about patient’s activity levels. We created a Wellness Score using proteins in 55 patient’s blood samples. This score groups patients into those who were likely to die and those who were likely to be alive within six months of their blood sample. This score was more accurately able to predict a patient’s survival at six months than PS. We then reached the same conclusion in a further 77 patients. We hope this score can now be tested in an even larger group of patients.

**Abstract:**

TARGET (tumour characterisation to guide experimental targeted therapy) is a cancer precision medicine programme focused on molecular characterisation of patients entering early phase clinical trials. Performance status (PS) measures a patient’s ability to perform a variety of activities. However, the quality of present algorithms to assess PS is limited and based on qualitative clinician assessment. Plasma samples from patients enrolled into TARGET were analysed using the mass spectrometry (MS) technique: sequential window acquisition of all theoretical fragment ion spectra (SWATH)-MS. SWATH-MS was used on a discovery cohort of 55 patients to differentiate patients into either a good or poor prognosis by creation of a Wellness Score (WS) that showed stronger prediction of overall survival (*p* = 0.000551) compared to PS (*p* = 0.001). WS was then tested against a validation cohort of 77 patients showing significant (*p* = 0.000451) prediction of overall survival. WS in both sets had receiver operating characteristic curve area under the curve (AUC) values of 0.76 (*p* = 0.002) and 0.67 (*p* = 0.011): AUC of PS was 0.70 (*p* = 0.117) and 0.55 (*p* = 0.548). These signatures can now be evaluated further in larger patient populations to assess their utility in a clinical setting.

## 1. Introduction

The development of personalised healthcare and targeted therapies requires the parallel development of companion biomarkers [1]. Such markers have great potential to be used in the personalised assessment of disease through prognostic, predictive or diagnostic means. Characterisation of patient selection biomarkers better informs the clinical decision both for clinical trials as well as for improving standard of care in medicine generally. Whilst genetic biomarkers and mRNA transcripts are typically analysed for patient selection, a majority of patients have no clear genomic driver of their disease. In contrast, proteins are the main target for many drugs and provide a higher amount of functional information and insight into cellular and organismal state compared to nucleic acid markers [2,3]. Thus, detailed proteomic analyses will provide insights for future patient selection to early phase clinical trials. The prognostic relevance of proteins is also important in helping to select patients to early phase clinical trials. The precedent for proteins as a prognostic biomarker is demonstrated, for example, by haemoglobin (Hb) and lactate dehydrogenase (LDH) that have already been used in determining health status and prognosis in patients prior to enrolment in early phase cancer clinical trials [4,5].

Performance status (PS) was developed 70 years ago to assess patient’s ability to perform day-to-day activities. The Eastern Cooperative Oncology Group (ECOG)/World Health Organisation (WHO) scale developed in 1982 is now the most widely used scale, scoring patients on a scale of 0 to 5. Scores are defined as in Table S1.

The subjective nature of the PS scale has led to recognition that there is a requirement for improvement in the early phase cancer clinical trial setting. Early phase cancer clinical trials are designed with the aim of identifying the side effects and correct dosage levels of a given investigational medicinal product (IMP) alongside initial efficacy evaluation. Patients considered for early phase cancer trials typically have advanced stage disease and have usually received prior therapies. Being able to predict which patients are fit enough for enrolment into a cancer clinical trial is important from a patient safety perspective and ensuring robust evaluation of the IMP. Early phase clinical trial inclusion criteria normally requires an acceptable PS score of either 0 or 1 and a life expectancy of >3 months [6]. Side effects are more life threatening to the patient volunteering for the trial with poorer PS [6]. Developing a more objective and quantitative tool than PS which is more suited to assessing patient status may enhance patient selection to early phase studies. Using new ‘omic’ approaches offers opportunity for inclusion of quantified biomolecules. Proteomic measures on peripheral blood have been studied as a means of assessing patient survival and health status [7,8,9]. In addition to using PS, routine measurement of albumin, LDH and Hb have been employed to assess patient wellness with well-studied correlations between circulating levels and patient survival [5,10,11,12]. Prognostic scoring systems have been previously developed using different methodologies and techniques. Examples of these scoring methods include the real-world prognostic score (ROPRO) [10] which was calculated using routine clinical variables from over 120,000 patients to provide impressive prognostic utility, the Royal Marsden prognostic score [11], the inflammation-based Glasgow prognostic score (GPS) [12] which uses albumin and C-reactive protein levels and the international Metastatic Renal-Cell Carcinoma Database Consortium prognostic model [13] which uses haemoglobin, calcium, neutrophil and platelet levels along with the Karnofsky performance status [14].

A multidisciplinary approach in biomarker discovery and validation provides a higher probability of identifying more accurate and sensitive tests [15]. Mass spectrometry (MS) based plasma proteomics can be employed to develop a wellness assessment [16]. The large number of proteins assessed using MS offers deep insight into the individual phenotype and has a level of specificity that is required in bioassays [17,18], especially when combined with an artificial intelligence based approach to data interrogation [19,20].

The wide heterogeneity of patient cancer phenotypes is a confounding factor in developing sensitive and specific algorithms for clinically relevant information to be derived. Plasma protein biomarkers can allow for the separation of patients into groups depending on their prognostic scoring. Proteomics has been shown to be a useful tool in early diagnosis [21,22], prediction of wellness [23], treatment allocation [24,25], disease progression and aetiology [26,27] as well as predicting drug resistance [24].

Sequential window acquisition of all theoretical fragment ion mass spectra (SWATH-MS) is a biomarker discovery tool that can be used to identify many proteins in a consistently reproducible way [28]. SWATH-MS uses the reproducibility and relatively swift sample preparation and run time plus standardised informatics capabilities to generate digitised proteomic maps. SWATH-MS has been used to identify proteomics signatures in a wide range of diseases and conditions with recent cancer based studies finding signatures of interest in breast and colorectal cancer [29,30], endometrial cancer [31], ovarian [32] lung cancer [33] and other health conditions such as weight loss [34].

SWATH-MS techniques generate fragment spectra from all MS measurable peptides that are within a sample allowing for the analysis of a wider range of typically low abundance biomarkers [35,36,37,38]. We have taken the SWATH-MS platform and applied it to samples collected as part of the TARGET (tumour characterisation to guide experimental targeted therapy) study. The TARGET trial is an initiative to optimise the pathway for molecular characterisation of all patients being considered for early phase cancer trials in order to inform clinical decisions as to their optimal treatment [39]. Patients are matched to relevant therapies in the early phase clinical trials on the basis of molecular screening and/or disease type. Nonetheless wellness to engage on a clinical trial is still relatively subjectively assessed with scoring algorithms lacking sensitivity [40,41]. Here we have taken 10 patient variables including standard prognostic parameters and assessed the plasma proteomic profile. With these data we sought to develop an improved prognostic algorithm. The aim of this study was therefore to identify potential proteomic biomarkers that have prognostic value in determining the wellness of patients enrolling into phase 0/I trials in a discovery and validation set in order to assess the capability of translating these markers into clinical utility.

## 2. Materials and Methods

### 2.1. Clinical Sample Collection

The TARGET trial was conducted in line with the principles of the Declaration of Helsinki and Good Clinical Practice. The trial was approved by the North-West (Preston) National Research Ethics Service, in February 2015 (15/NW/0078) and is registered in the NIHR Central Portfolio Management System (CPMS ID 39172). All patients were recruited by the Experimental Cancer Medicine Team at The Christie NHS Foundation Trust, Manchester, UK. All patients provided fully informed written consent for provision of tumour and blood samples and clinical data.

The study design and eligibility criteria have been described previously [36]. Briefly, the TARGET trial was split into two parts. In part one, the aim was to assess the ctDNA and tumour sequencing workflow and its capabilities in stratifying patients. In part two, the aim was to expand the study in order to stratify patients into clinical trials and therapies in real time. For this investigation, 73 patients were used as the discovery cohort and 79 patients used as the validation cohort.

### 2.2. Proteomic Sample Collection and Preparation

Double-spun plasma samples were collected using 10 mL EDTA tubes. Blood was spun within 96 h of collection prior to storage at −80 °C. Plasma samples taken at the patient’s baseline visit for enrolment into the TARGET trial were used for this study. For the discovery phase of the study, 73 patient samples were used, however 18 samples did not pass quality checks (e.g., due to haemolysis or presence of cell lysis products) and these patients were not included in the analysis. For the validation phase of the study, 2/79 samples did not pass quality control filters. Our workflow only assesses tryptic peptides in order to avoid endogenous protease activity affecting our quantification. Furthermore, we have assessed leaving plasma samples on a lab bench at room temperature for varying levels of time and have observed no effect on the relative abundance levels of the proteome.

Plasma samples were depleted of the top 12 most abundant proteins using the commercially available Top-12 kits (Pierce, Thermo, Loughborough, UK) according to the manufacturer’s methods. The resultant solution was assayed for the protein amount using a Bradford reagent (Bio-Rad, Watford, UK). Solution containing 40 μg worth of protein was taken and processed further. Samples were reduced using 60 mM TCEP at 60 °C for 60 min followed by alkylation using 10 mM iodoacetamide in the dark for 30 min. Digestion was performed using trypsin (Promega, Southampton, UK) overnight at 37 °C in a 10:1 ratio of protein to enzyme. Digested peptides were cleaned using a SepPak (Waters, Wilmslow, UK) 96 well plate SPE system.

### 2.3. SWATH-MS Analysis

Mass spectrometry was performed using a 6600 TripleTOF (Sciex, Warrington, UK). The LC method was a 120-min gradient between a buffer A of 98% Water, 2% (*v/v*) Acetonitrile and 0.1% (*w/v*) Formic Acid and a buffer B of 80% Acetonitrile, 20% Water, 0.1% Formic Acid. Samples were injected in duplicate. In the discovery set, a Dionex Ultimate 3000 HPLC was connected in-line (Dionex, Thermo, Loughborough, UK) and the peptide samples were loaded onto a trap column, 5 μm C18 PepMap 100 (Thermo, Loughborough, UK), for 10 min at 5 μL/min before loading onto an Acclaim C18 PepMap 100 analytical column at 300 nL/min (Thermo, Loughborough, UK). In the validation set, an Eksigent system comprising of a nanoLC 400 autosampler along with a 425 pump module were used with a YMC-Triart C18 trap column and a YMC-Triart C18 analytical column. Spectra were acquired in a SWATH mode method utilising the 100 variable window method with MS2 windows ranging from 399.5 to 1249.5 *m/z* with optimised collision energy equations. MS1 mass range was between 100–1500 *m/z* with an accumulation time of 0.05 s and a cycle time of 2.6 s.

Spectral data files were converted using wiffconverter (Sciex, Warrington, UK) to mzML format prior to search using openSWATH (Version 2.0.0) against a the publically available twin plasma library (version published 5 January 2015) [42]. openSWATH results files were processed using pyProphet before being aligned using the feature alignment script from MSproteomicstools with a target FDR of 0.01 at the PSM level. Data are available via ProteomeXchange with identifier: PXD023553.

### 2.4. Statistical Analysis

Data analysis was performed using R (Version 3.4.1) and the IBM Statistical Product and Service Solutions (SPSS) (Version 25). For SWATH-MS data in both the discovery and validation cohorts, the Bioconductor (Version 3.5) packages MSstats and SWATH2Stats were used for downstream processing. Coefficients of variation were calculated between technical replicates with any samples showing a median CV of 20% or higher being re-run, this resulted in 15 samples being re-injected. The median CVs of all samples ranged from 5.6% to 18.9% with a median of 7.9%. An example correlation between two technical replicates is shown in Supplemental Figure S1. Data was filtered by mscore using the filter_mscore_fdr function in the SWATH2stats package with an overall protein FDR target of 0.02 and an upper overall peptide FDR limit of 0.05. Data was converted from a feature alignment output to MSstats input using SWATH2stats’s convert4MSstats function. MSstats was used for normalisation and summarisation of protein intensities using the dataprocess function utilising the default arguments. Missing values were imputed using the Muiltivariate Imputation by Chained Equations (MICE) package (Version 2.3) using only proteins seen in 70% of the samples. The proteins verses samples matrix was imputed using the default arguments of the mice function with the predictive mean matching method and the random seed set to 500. Significance between protein abundances were calculated using the Limma package using the empirical Bayes statistics for differential expression method. Significance was set as *p* < 0.05, 95% confidence intervals were determined where necessary as advised in text. RandomForest was employed by separating the discovery data into training and testing sets at a 70% split. A total of 1000 models were created and the protein importances were ranked across all models. Accuracy was used as the parameter to optimise. For categorical variables, a value of 0 was allocated to the favourable category and a 1 for not favourable. Univariate and multivariate analysis was evaluated using Cox regression. Overall survival was measured from the date of consent to TARGET to date of death of any cause. To determine the impact of all the collected variables in the demographic characteristics on the patient’s allocation to the wellness score (WS) groups, a two-sided Fisher’s exact tests were used in groups with fewer than five patients and a Pearson’s Chi squared tests were utilised when there were more than five patients in a group. Overall survival was determined using Kaplan–Meier curves. ROC curves and AUC were used to determine the prognostic abilities of the scores created. Evaluation of the prognostic biomarkers was performed taking into account the REporting recommendations for tumour MARKer prognostic studies (REMARK) guidelines [38].

### 2.5. Clinical Prognostic Parameters

Full clinical patient demographics and clinical characteristics can be found in Table S3 along with a boxplot showing the distribution of patients with each PS (Figure S2). Lines of treatment were considered new when they were given after documented clinical or radiological disease progression. Any treatment given to cure disease, for example surgery or to slow progression were counted as treatment. If treatment was only given for symptom control (e.g., palliative radiotherapy), this was not counted. Number of treatment lines was used to determine how heavily pre-treated patients had been. Sites of disease were identified using the patient’s most recent computed tomography (CT) scan taken before they enrolled into TARGET. Sites of disease were counted if there was disease present at the time of the scan. If a site of disease had been removed via surgery and had not recurred, it was not counted. An ‘above diaphragm’ and ‘below diaphragm’ was used for counting lymph nodes, the maximum number of lymph nodes counted as a site per person was two. Patients with one site of disease were allocated a 0 and patients with greater than one site of disease were allocated a two.

Data for circulating blood levels of albumin, LDH and Hb were collected on all patients in both cohorts upon entry into the TARGET trial because they have been used previously as prognostic biomarkers alongside PS [10]. Poor prognostic cut-offs were defined as low serum albumin less than 35 g/L (Figure S3); high LDH > 500 IU/L (Figure S4) and low Hb (anaemia), <115 g/L (Figure S5).

Patient overall survival was calculated from the date of consent to the TARGET trial. Patient survival status was accurate as of 10 May 2019.

## 3. Results

### 3.1. Determination of a Discriminatory Panel

Using SWATH-MS we derived a proteomic map of all the plasma samples in the discovery set. In total 995 proteins were identified (>1 proteotypic peptide, Tables S5 and S6). The results had a mean assay FDR of 0.14; 550 proteins were seen in >70% of all samples. A total of 77 proteins were significantly (*p* ≤ 0.05) different between samples from patients who died within 6 months of plasma sampling and those who died beyond 6 months. Machine learning techniques were utilised to identify candidate proteins to assess further in downstream statistical analysis. SVC, K Nearest neighbour and RandomForest analyses were employed on all the proteins. RandomForest results created the panel with the strongest discrimination. A shortlist of 15 proteins were isolated as providing the best differentiation.

**Figure 1:**
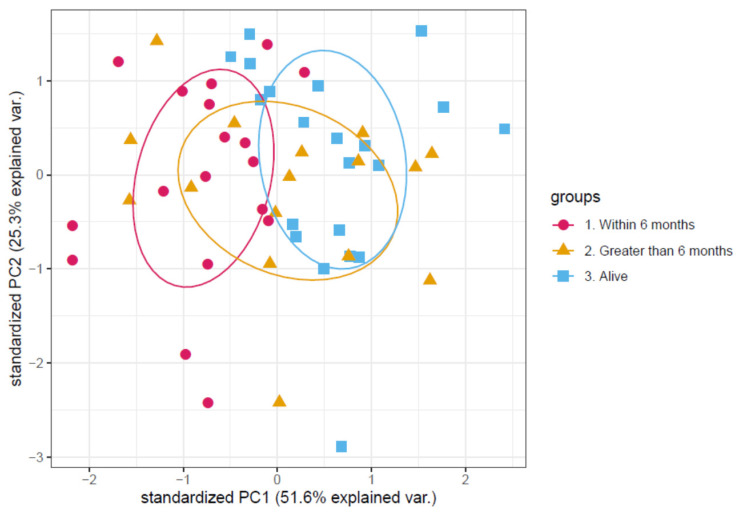
Principal component analysis of the protein abundance levels of the final panel of proteins. Each point in the principal component analysis is an individual patient sample. Patients are colour coded according to the patient’s survival time after samples were taken. With those still alive at time of analysis coloured in blue, those who survived longer than 6 months coloured in orange and those who died within 6 months coloured in magenta. There is a separation between those that died within 6 months and the remainder of the samples along the first principal component with an explained variation of 51.6%.

This list of proteins was then filtered down further by Cox regression analysis which determined the significance of each protein on overall survival (OS) (Table 1). For a clinically viable assay the number of proteins for measurement needs to be limited. Three proteins of interest had a significant correlation with overall survival. A PCA (Figure 1) showed separation between the different experimental groups using this shortlist of three proteins. Within the PCA the different experimental groups separated on the first principal component with an explained variation of 51.6%. One protein showed a positive correlation, and two proteins showed a negative correlation (positive correlation was increased amount of protein correlated with an increased risk of death over time from consent to the last time point the patient was seen alive). The protein with a positive correlation was Leucine-rich alpha-2-glycoprotein. The two proteins with a negative correlation were Apolipoprotein C-III and Plasma serine protease inhibitor.

**Figure 2:**
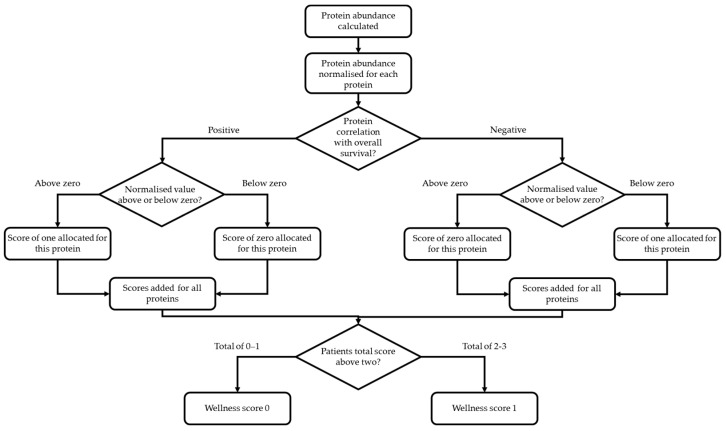
Decision tree for calculation of the Wellness Score for all patients. After protein abundances were calculated from mass spectrometry data, they were individually normalised. A zero value was provided to signify a decrease in the risk of death. If a normalised value of a positively correlated protein was above zero a value of one was given and a value of zero given if the normalised value was below zero. If a normalised value of a negatively correlated protein was above zero a value of zero was given and a value of one given if the normalised value was below zero. For each patient, their protein scores were summed together. The Wellness Score was categorised as 0 if they had a total summed protein score of 0–1 and a Wellness Score of 1 if they had a total summed protein score of 2–3.

In order to create a Wellness Score using all three proteins, a number of approaches were used consistent with development of a meaningful clinical tool. This decision process is shown in Figure 2. Zero was used to signify a decrease in risk of death.

### 3.2. Validation of the Proteomic Signature

Using SWATH-MS on the validation cohort 1089 Proteins were identified that had at least one proteotypic peptide identified (Table S7). The mean assay FDR of all the samples was 0.08 with 885 proteins seen in at least 70% of the samples. A total of 118 proteins were significantly (*p* < 0.05) different between patients who died within 6 months of plasma sampling and those who died beyond 6 months.

**Figure 3:**
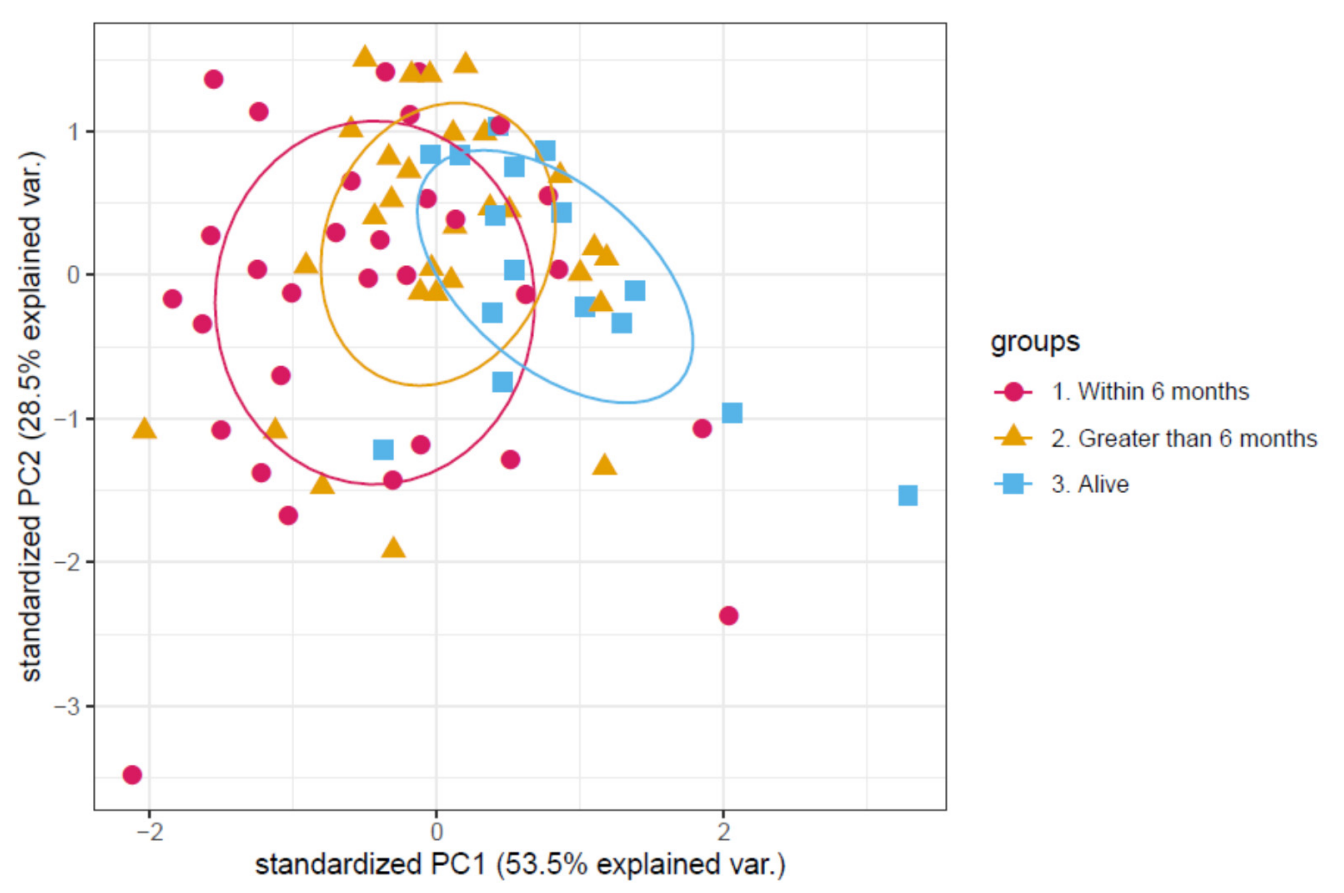
Principal component analysis of the protein abundance levels of the validation set. Each point in the principal component analysis is an individual patient sample. Patients are colour coded according to the patient’s survival time after samples were taken. With those still alive as of publication coloured in blue, those who survived longer than 6 months coloured in orange and those who died within 6 months coloured in magenta. There is a slight separation visible between those that died within 6 months and those that died after 6 months.

Using the same panel of three proteins identified in the discovery cohort, the validation cohort was assessed for a similar effect on overall survival. The PCA is shown in Figure 3. Univariate Cox regression analysis on each of the proteins showed that three proteins showed a significant impact on survival: Leucine-rich alpha-2-glycoprotein (positively correlated with a *p* value of 2.0 × 10^−4^), apolipoprotein C-III (negatively correlated with a *p* value of 0.021) and plasma serine protease inhibitor (negatively correlated with a *p* value of 0.0001). All three proteins showed the same direction of correlation as in the discovery cohort. Thus, we have validation of the plasma proteome analysis.

### 3.3. Assessment of Prognostic Scoring Methods

Within the discovery set, univariate Cox regression analysis was performed on all of the characteristics listed in Table S2. Of these, Wellness Score, PS, and the number of sites of disease were all significantly correlated with overall survival (with *p* values of 0.000551, 0.001, and 0.007 respectively) with the remaining variables showing no significant regression with overall survival. Within the validation cohort, Wellness Score, albumin, LDH, Hb and the number of disease sites were all seen as significant (*p* values of 0.000451, 0.000437, 0.001, 0.000002, and 0.011 respectively). A chi-squared test was performed between the PS score and Wellness Score in the discovery and validation set showing a non-significant correlation in both (*p* = 0.104 and *p* = 0.699). In both data sets, Fisher’s exact tests showed a significant correlation between survival status and wellness score (*p* = 0.006 and *p* = 0.001). In the validation set, chi-squared test showed a significant correlation between Wellness Score and baseline LDH (*p* = 6.3 × 10^−5^).

The variables found to be significant at univariate analysis were put in a multivariate analysis for both data sets. The *p* values for Wellness Score, sites of disease and PS were *p* = 4.0 × 10^−5^, *p* = 0.021 and *p* = 0.001 in the discovery cohort and *p* = 6.5 × 10^−5^, *p* = 0.004 and *p* = 0.044 in the validation cohort at multivariate analysis. Although PS was not found to be significant in the validation set, as it was found to be significant at multivariate analysis, it was taken forward for score creation. Two more scores were generated for comparison, the first being an Enhanced Proteomics PS Score (PEPS). This was calculated by adding together the Wellness Score (0 for low and 1 for high) and PS (0 for PS 0 and 1 for PS 1–2) to create a score out of 2. The third score generated was a Phase I Proteomics score (PPM) by adding in variables that were significantly related to overall survival in the multivariate analysis. With a single disease site being represented as a 0 and multiple sites represented as a 1. PS was represented as before in the PEPS. The addition of the disease site value to the PEPS gave a PPM score out of 3. Score allocation summaries can be found in Table S3.

**Figure 4:**
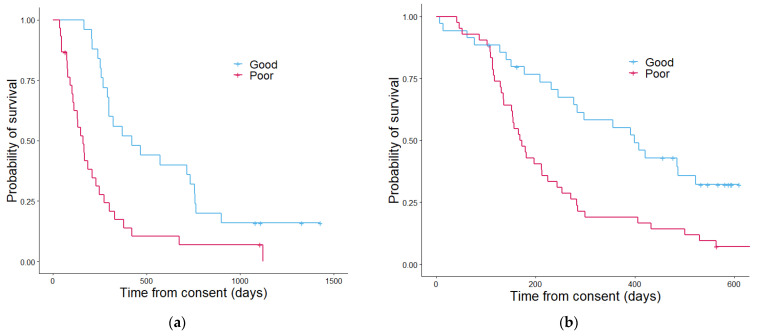
Kaplan–Meier curves showing the overall survival determined by the Wellness Score in the discovery cohort (**a**) and the validation cohort (**b**). In the discovery cohort (**a**) patients with a good outcome Wellness Score had a median overall survival of 422 days and patients with a poor outcome Wellness Score had a median overall survival of 161 days. The *p* value was 5.5 × 10^45^. In the validation cohort (**b**) the median overall survival estimate in days for patients with a good outcome Wellness Score was 407 and 167 for patients with a poor outcome Wellness Score. The *p* value was 4.5 × 10^−4^.

Kaplan–Meier analysis was used to estimate the survival function when stratifying patients using each of the different scores generated. The Wellness Score approach (Figure 4) showed a significant difference in overall survival between the those with a good outcome Wellness Score and those with a poor outcome score with regards to overall survival in both the discovery cohort and validation cohort (*p* = 5.5 × 10^−4^ and 4.5 × 10^−4^ respectively). The number of patients separating into each Wellness Score group and survival status showed a similar pattern between discovery and validation (Figure S6). In the discovery cohort positive predictive value (PPV) of the Wellness Score of overall survival at six months was 96% and negative predictive value (NPC) of 60% (Table 2). The validation set had a PPV of 74% and a NPV of 60%. The Wellness Score was able to accurately predict the outcomes at six months of 75% of patients in the discovery set and 66% in the validation. PS (although not a tool created to predict patients’ outcomes at six months) had a PPV of 47% in the discovery cohort, with an NPV of 60%. In the validation cohort, PS PPV was 54% and NPV 59%. In respect of early phase clinical trials activity it would appear a Wellness Score could be useful for enrolment in such clinical trials by adding a further means of stratification, as the PPV of WS in greater than PS at predicting if a patient will be alive in six months. As using PS score alone had a lower predictive capability, the use of a combined score comprising of PS, the number of disease sites and Wellness score was investigated in a combined Phase I proteomics score PPM score.

As seen in Table 3 we now have shown a significant difference between the two stratified patient groups regarding overall survival in the discovery cohort as well as the validation cohort (*p* = 4.4 × 10^−6^ and 7.5 × 10^−5^ respectively), using proteomics to derive markers.

**Figure 5:**
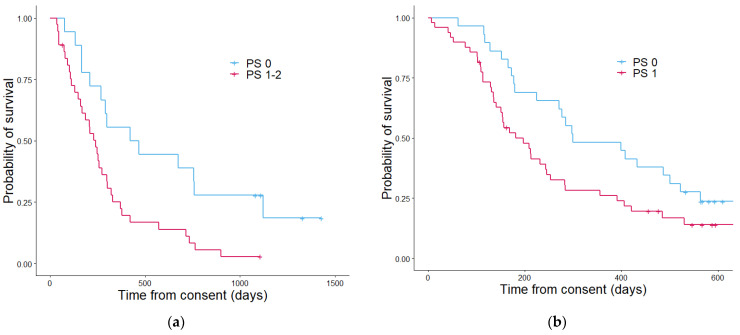
Kaplan–Meier curves showing the overall survival determined by the performance status in the discovery cohort (**a**) and the validation cohort (**b**). In the discovery cohort (**a**) patients with a PS score of 0 had a median estimated overall survival in days of 628 and patients with a PS score of 1–2 had a median overall survival of 296 days. Table 3. In the validation cohort (**b**) the median overall survival estimates in days patients with a PS of 0 was 298 and for PS score 1–2 was 196. The *p* value was 0.084.

**Figure 6:**
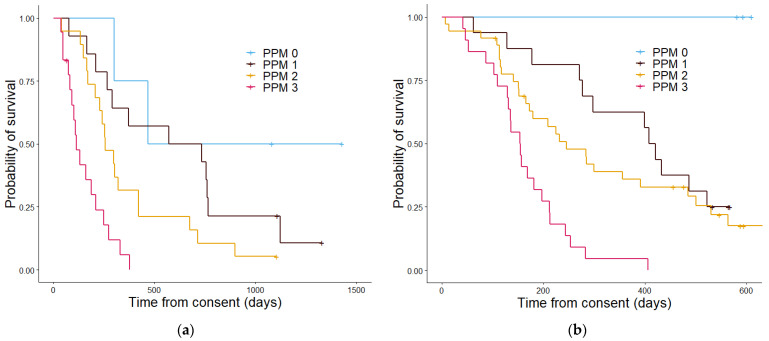
Kaplan–Meier curves showing the overall survival determined by the Proteomics Score in the discovery cohort (**a**) and the validation cohort (**b**). In the discovery cohort (**a**) patients with a PPM score of 0 had a median estimated overall survival in days of 467, patients with a PPM score of 1 had a median overall survival of 612 days, patients with a PPM score of 2 had an estimated median overall survival of 229 days, and patients with a PPM score of 3 had median overall survival of 148 days. The *p* value was 4.42 × 10^−6^. In the validation cohort (**b**) the median overall survival estimate in days for PPM score 0 could not be calculated as all patients in this group were alive, for PPM score 1 the median overall survival in days was 407, patients with a PPM score 2 had an estimated median overall survival of 283 days and those with a PPM score 3 had an median overall survival of 153 days. The *p* value was 7.4 × 10^−7^.

**Figure 7:**
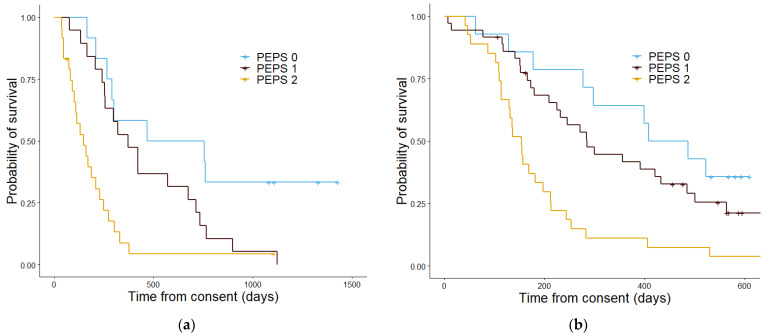
Kaplan–Meier curves showing the overall survival determined by the proteomics enhanced performance status in the discovery cohort (**a**) and the validation cohort (**b**). In the discovery cohort (**a**) patients with a PEPS score of 0 had a median estimated overall survival in days of 612, patients with a PEPS score of 1 had an estimated median overall survival of 371 days and patients with a PEPS score of 2 had a median overall survival of 148 days. The *p* value was 5.3 × 10^57^. In the validation cohort (**b**) the median overall survival estimate in days patients with a PEPS of 0 was 486, for PEPS score 1 was 298, and for PEPS score 2 was 153. The *p* value was 7.4 × 10^−7^.

PS (Figure 5) was not able significantly stratify patients in the validation cohort (*p* = 0.084) but was able to in the discovery group (*p* = 0.001). PPM (Figure 6) also showed a significant difference in stratifying patients into three groups, within the discovery (*p* = 4.4 × 10^−6^) and validation cohorts (*p* = 7.4 × 10^−7^). PEPS (Figure 7) showed a significant difference between the two stratified patient groups regarding overall survival in the discovery cohort and in the validation cohort (*p* = 5.3 × 10^−5^ and 7.5 × 10^−5^ respectively).

**Figure 8:**
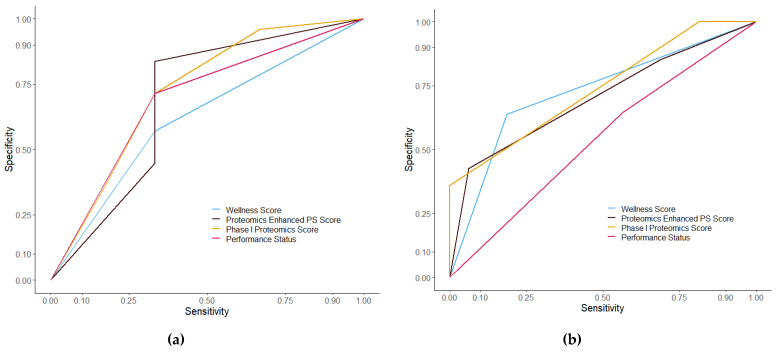
ROC curves comparing the different prognostic scores in the discovery cohort (**a**) and the validation cohort (**b**). The different scoring systems were analysed using receiver operating characteristics curves. In the discovery cohort (**a**) the AUC for PS score was 0.690 with a *p* value of 0.131, the AUC for PPM score was 0.832 with a *p* value of 0.008, the AUC for PEPS score was 0.810 with a *p* value of 0.014, and the AUC for Wellness Score was 0.755 with a *p* value of 0.043. In the validation cohort (**b**) the AUC for PS score was 0.549 with a *p* value of 0.548, the AUC for PPM score was 0.745 with a *p* value of 0.003, the AUC for PEPS score was 0.702 with a *p* value of 0.013, and the AUC for Wellness score was 0.726 with a *p* value of 0.006.

**Figure 9:**
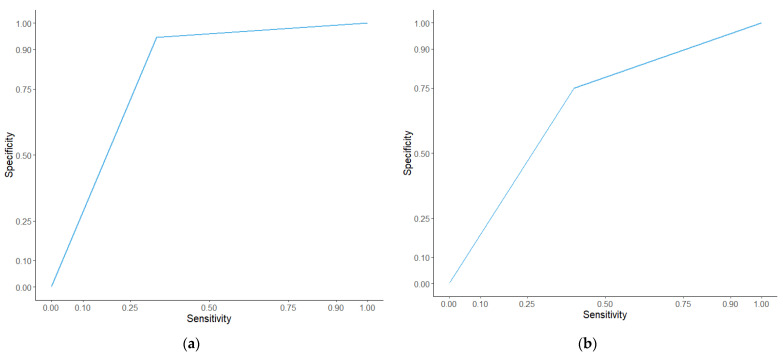
ROC curves of the Wellness Score in the discovery cohort (**a**) and the validation cohort (**b**) at six months. The Wellness Score was analysed using receiver operating characteristics curves. In the discovery cohort (**a**) the AUC for Wellness Score was 0.756 with a *p* value of 0.002. In the validation cohort (**b**) the AUC for Wellness Score was 0.675, with a *p* value of 0.018.

The difference scores were assessed using receiver operatic characteristics curves (Figure 8). The false positive rate, or specificity, was plotted against the true positive rate, or sensitivity. Wellness Score had an AUC of 0.755 with a *p* value of 0.043 in the discovery cohort and an AUC of 0.713 with a *p* value of 0.009 in the validation cohort. The PS score had an AUC of 0.697 with a *p* value of 0.117 in the discovery cohort and an AUC of 0.549 with a *p* value of 0.548. PPM score had an AUC of 0.832 with a *p* value of 0.008 in the discovery cohort and an AUC of 0.745 with a *p* value of 0.003 in the validation cohort. PEPS score had an AUC of 0.810 with a *p* value of 0.014 in the discovery cohort and an AUC of 0.733 with a *p* value of 0.004 in the validation cohort. The different scores, as predictors, were then tested at different time points prior to death. ROC analysis showed an AUC of 0.756 and a *p* value of 0.002 at six months from consent (Figure 9) and an AUC of 0.733 and a *p* value of 0.003 at nine months in the discovery set. Other time periods were tested but showed a less significant model than nine months (Table S4). A direct comparison with published prognostic scoring systems was not immediately possible given the patient data obtained from patients entering the TARGET trial. A comparison with GPS was possible using the proteomic data obtained through SWATH-MS and the levels of albumin recorded upon patient consent. To make the GPS modified score, SWATH-MS determined protein abundance levels of C-reactive protein (CRP) were normalised. Patient samples with a CRP normalised abundance greater than 0 were given a CRP score of 1 and all those with a normalised abundance below 0 got a CRP score of 0. Albumin was divided into <35 g/L = 1 and >35 g/L = 0. These were then added together to make a GPS modified score. No patient sample in the discovery set had both a high CRP score and a low albumin score so no patients had a GPS modified score of 2 and only one patient sample in the discovery set had an albumin of <35 g/L. Kaplan–Meier analysis of the modified GPS (Figure S7) showed no significant difference in overall survival between scores in the discovery set but found a significant difference in the validation set (*p* = 0.774 and 2.36 × 10^−7^ respectively). A ROC curve of the modified GPS was plotted in addition to the curves in Figure 8, shown in Figure S8. The two patient cohorts showed a disproportionate amount of colorectal cancer patients compared to other cancer types, considering that leucine-rich alpha-2-glycoprotein was a constituent part of the protein panel the Kaplan–Meier analysis on the Wellness Score was repeated but with the colorectal cancer patients excluded from the analysis (Figure S9). The analysis still showed a significant difference in overall survival between those with a good outcome Wellness Score and a poor outcome Wellness Score in both the discovery and validation cohorts (*p* = 0.005 and 0.0144 respectively).

## 4. Discussion

Cancer patients enrolling into early phase clinical trials are a heterogenous group having a diverse range of cancer types and treatment histories. To ensure successful trials and to limit the unnecessary exposure of patients to treatments which they stand little chance of benefiting from as their disease is progressing too fast, a robust and reliable means of determining patient prognosis is needed. PS is one of the measures routinely used for determining whether patients are eligible for enrolment into early phase clinical trials. Many trials have a restriction of an expected life expectancy of 3–6 months required for patients to join the trial [39]. The subjective nature of PS allows for bias to be introduced from either the patient or the person assessing them. A routine blood protein test could provide a more objective means to determine the wellness of patients, their likely prognosis and their suitability for trials.

The proteins identified in our study have been seen previously with biological links to cancer. Leucine-rich alpha-2-glycoprotein has been used recently as a potential diagnostic marker in colorectal cancer [40] and it has shown potential use as a prognostic marker and treatment target in oesophageal squamous cell carcinoma [41]. A high amount of leucine-rich alpha-2-glycoprotein has been shown to promote angiogenesis which could indicate an increased amount of cancer invasion and metastasis [43]. Apolipoprotein C-III and other apolipoproteins have been identified as being significantly abundant in liver and lung cancer and the protein family has been identified as a potential target for diagnostic and prognostic markers [42]. A low level of apolipoprotein C-III would indicate a lower capability of the body to inhibit fat degradation and low levels of apolipoprotein C-III are observed in gastric cancer where blood lipid levels are correlated with disease progression [44]. Plasma serine protease inhibitor has been shown to have links with lung and ovarian cancer with the potential use as a prognostic marker [43,44]. A plasma serine protease inhibitor decrease is association with cancer metastasis, migration and invasion [45,46]. While the proteins in the panel in the Wellness Score have been linked with individual cancer types before in prognostic capacity, the use of these proteins in defining the wellness of patients across a wide range of cancers as experienced in early phase cancer clinical trials has not been identified previously.

In our initial discovery phase of the study, we were successful in identifying protein biomarkers that may have the potential for later development into a routine assay that facilitates appropriate enrolment into clinical trials whilst being sufficiently tractable to enable its usage to characterise patient wellness prior to starting a clinical trial using the Wellness Score. The significant results of this score were replicated in the validation cohort. This has demonstrated the capabilities of using proteomics to discover novel prognostic biomarkers for the stratification of patients into those whose disease is advancing so rapidly that there is only a short window for new investigational medicinal products to demonstrate efficacy. This is particularly pertinent for those investigational medicinal products which may have delayed responses—such as the immune-modulating class of drugs. The successful discovery of a set of protein biomarkers highlights the ability of discovery techniques such as SWATH-MS to find previously unknown biomarkers. Data-independent mass spectrometry analysis has gone through rapid development since its inception into proteomics. Recent work by the Markus Ralser group [47] has shown that it is possible to create ultra-high throughput workflows with sample to sample run-times of 5 min. SWATH-MS has been found to be robust in terms of reproducibility and quantitative variability between different instruments and laboratory sites [48]. While SWATH-MS presents an attractive option for use in a clinical setting in the future, presently more appropriate to generate assays utilising established methods of widespread routine clinical analysis. Single-reaction monitoring (SRM) based mass spectrometry along with antibody based quantitative methodologies such as ELISAs have long been established as routine tools in clinical laboratories [32]. The framework and experimental paradigm presented in this study, using SWATH-MS based proteomics along with statistical analysis to generate scoring systems, can be utilised further in different diseases and tissue types.

ROC curves of the different scores between the two cohorts showed that all the scores (except for PS in the discovery cohort) were accurate predictors in both the discovery cohort and in the validation cohort. The Wellness Score alone was a significant means to estimate overall survival in both cohorts. A comparison with the Glasgow prognostic score showed that Wellness Score provided stronger results indicating it could have the potential to be developed into a test that may be applicable to clinical settings in centres that are able to undertake early phase clinical trials. While a direct comparison against other prognostic scores is not possible due to the clinical data available, in comparison of model performance, the ROPRO score, calculated using information from more than 120,000 patients provided a high AUC value than the Wellness Score. As more samples become available where outcome data is available on sufficient patients, we will analyse the value of the protein signature using orthogonal methods more applicable to the selection of patients who are sufficiently well and able to enrol in a clinical trial. When the Wellness Score was enhanced with additional metrics it provided more confident results. PS alone was not such a reliable means of assessing patients with regards to overall survival but was useful in increasing the strength of the Wellness Score. While the three different scores all were useful predictors of overall survival, the Wellness Score has an advantage over the others in our aim to potentially produce a more objective scoring system in the future, based upon the Wellness Score, in a clinical setting. PPM score includes the amount of disease sites which is a complex and subjective metric that is not routinely collected by clinical trials. PEPS includes PS which is based upon subjective assessment. Therefore, the Wellness Score is the most appropriate to be followed in further, future studies.

A limitation of this study is the low number of patients. Larger scale testing of the Wellness Score would be needed before translation into a clinical setting. The data collection window for the validation set was shorter than the discovery set. Having the same data collection period after the first patient had consented would have given more comparable data. Another limitation is the lack of healthy normal controls which would be needed to assess whether the Wellness Score is influenced by cancer. A limitation of study design with respect to machine learning analysis is that there were two patients with PS score 2 in the discovery set and none in the validation set, and therefore introducing bias into the models. While the proteomic based scoring systems in this study have been shown to provide stronger correlates with patient overall survival than PS, they are also more invasive and costly than PS to determine. Using antibody-based techniques with highly specific antibodies to each of the three identified proteins may provide greater sensitivity as prognostic biomarkers. Exploration of the relative contribution of each protein to a composite score, and whether one protein alone would provide sufficient clinical utility could also be explored in such a confirmatory study. A comparison should also be made to established prognostic tools currently in use in this patient population such as the Royal Marsden prognostic score (RMH Score) [45].

## 5. Conclusions

In this study we assessed the prognostic value of a derived proteomic signature and found that such a signature had use as a means of determining the appropriate enrolment of patients into early phase trials. We have shown that proteomics enhanced PS, and other means that we have assessed, have a higher discriminatory capability than the standard metric of PS by combining the existing assessments with SWATH-MS proteomic data. The Wellness Score created in this study can potentially provide a more objective prognostic scoring that one that can differ depending upon the assessor. While integrating patient assessment and clinical observation can provide a stronger predictor of overall survival, they cannot be translated into a routine clinic-based test. We can now verify the value of this approach and assess the use of these proteins more generally as markers for cancer. Specifically, we have identified three proteins, previously associated with cancer which warrant further investigation as to their clinical utility (either alone or in combination) to predict overall survival more accurately as a prelude to incorporating them into patients’ pre-trial screening procedures for their eligibility to enrol into an early phase cancer clinical trial.

## Figures and Tables

**Table 1:** Proteins showing a significant (*p* < 0.05) Cox regression against overall survival along with hazard ratios and confidence intervals (CI).

Protein Name (Correlation)	*p* Value	Hazard Ratio	Lower 95% CI	Upper 95% CI
A2GL (+)	0.001	2.328	1.394	3.887
APOC3 (−)	0.023	0.7	0.514	0.952
IPSP (−)	0.013	0.437	0.227	0.84

**Table 2:** Positive and negative predictive values of Wellness Score and PS in the discovery and validation cohorts at six months.

Predictive Values	Discovery	Validation
WS PPV	96%	74%
PS PPV	47%	54%
WS NPV	60%	59%
PS NPV	60%	59%

**Table 3:** Median overall survival (OS) and significance of Wellness Score, Enhanced Proteomics PS score (PEPS), Phase I proteomics score (PPM), and Performance Status (PS) in the discovery and validation cohorts.

Scoring System	Score	Discovery *n*	Discovery Median OS (Days)	Validation *n*	Validation Median OS (Days)
Wellness Score	0	34	377	35	407
	1	21	148	42	167
	*p* value		5.5 × 10^−4^		4.5 × 10^−4^
PPM	0	6	1052	3	593
	1	16	572	16	407
	2	18	257	36	283
	3	15	112	22	153
	*p* value		4.4 × 10^−6^		7.4 × 10^−7^
PEPS	0	16	467	14	486
	1	20	377	36	298
	2	19	130	27	153
	*p* value		5.3 × 10^−5^		7.5 × 10^−5^
PS	0	18	628	29	298
	1 (+2)	37	296	49	196
	*p* value		0.001		0.084

## Data Availability

Data are available via ProteomeXchange with identifier: PXD023553.

## Supplementary Materials

The following are available online at https://www.mdpi.com/article/10.3390/cancers13102443/s1, Figure S1: Exemplar correlation plot between two technical replicates, Figure S2: Performance status and time until death in the two cohorts, Figure S3: Albumin levels compared against the days until patient death, Figure S4: LDH levels compared against the days until patient death, Figure S5: Hb levels compared against the days until patient death, Figure S6: Stacked bar charts showing the proportion of patients stratified by Wellness score and survival status at 6 months in the discovery cohort and the validation cohort, Figure S7: Kaplan–Meier curves showing the overall survival determined by modified GPS in the discovery cohort (A) and the validation cohort (B), Figure S8: ROC curves comparing the different prognostic scores along with modified GPS in the discovery cohort (A) and the validation cohort (B), Figure S9: Kaplan–Meier curves showing the overall survival determined by wellness score with colorectal patients excluded in the discovery cohort (A) and the validation cohort (B), Table S1: ECOG Performance Score categories, Table S2: Summary of cancer types within the discovery and validation samples, Table S3: Patient demographics and clinical characteristics, Table S4: Summary of AUC values from ROC analysis for Wellness score as a predictor of death in discovery set, Table S5: List of identified peptides and associated proteins within the discovery cohort, Table S6: List of the proteins and associated normalised relative abundances within the discovery cohort, Table S7: List of proteins and associated normalised relative abundances within the validation cohort.
