# Supplementary material for: Discovery and Evaluation of Protein Biomarkers as a Signature of Wellness in Late-Stage Cancer Patients in Early Phase Clinical Trials
Source: Cancers (Basel). 2021 May 18;13(10):2443. doi: 10.3390/cancers13102443 (PMC8157875; doi:10.3390/cancers13102443)
Supplement: Supplementary file 1 [file cancers-13-02443-s001.zip › cancers-1203252-SI.pdf]

cancers

MDPI

Article

Discovery and Evaluation of Protein Biomarkers as a Signature of Wellness in Late-Stage Cancer Patients in Early Phase Clinical Trials

Bethany Geary [1,2], Erin Peat [3], Sarah Dransfield [3], Natalie Cook [3], Fiona Thistlethwaite [2,3], Donna Graham [3], Louise Carter [2,3], Andrew Hughes [2], Matthew G. Krebs [2,3,*] and Anthony D. Whetton [1,2,4,*]

1 Stoller Biomarker Discovery Centre, Faculty of Biology, Medicine and Health, University of Manchester, Manchester, M13 9NQ, UK; bethany.geary@manchester.ac.uk
2 Division of Cancer Sciences, Faculty of Biology, Medicine and Health, University of Manchester, Manchester M13 9PL, UK; fiona.thistlethwaite@nhs.net (F.T.); louise.carter24@nhs.net (L.C.); Andrew.hughes@manchester.ac.uk (A.H.)
3 The Christie NHS Foundation Trust, Manchester Academic Health Science Centre, Manchester, M20 4BX, UK; erin.peat@postgrad.manchester.ac.uk (E.P.); sarah.dransfield@christie.nhs.uk (S.D.); natalie.cook17@nhs.net (N.C.); donna.graham8@nhs.net (D.G.)
4 Manchester National Institute for Health Research Biomedical Research centre, Manchester, M13 9WL, UK
* Correspondence: matthew.krebs@manchester.ac.uk (M.G.K.); tony.whetton@manchester.ac.uk (A.D.W.); Tel.: +44-(0)161-275-6267 (A.D.W.)

Citation: Geary, B.; Peat, E.; Dransfield, S.; Cook, N.; Thistlethwaite, F.; Graham, D.; Carter, L.; Hughes, A.; Krebs, M.G.; Whetton, A.D. Discovery and Evaluation of Protein Biomarkers as a Signature of Wellness in Late-Stage Cancer Patients in Early Phase Clinical Trials. *Cancers* **2021**, *13*, 2443. https://doi.org/10.3390/cancers13102443

Academic Editors: Udayan Guha and Xu Zhang

Received: 14 April 2021
Accepted: 12 May 2021
Published: 18 May 2021

Publisher's Note: MDPI stays neutral with regard to jurisdictional claims in published maps and institutional affiliations.

Copyright: © 2021 by the authors. Licensee MDPI, Basel, Switzerland. This article is an open access article distributed under the terms and con-

ditions of the Creative Commons Attribution (CC BY) license (http://creativecommons.org/licenses/by/4.0/).

Table S1. ECOG Performance Score categories [46,47].

PS Score	Patient Mobility
0	Fully active, able to carry on all pre-disease performance without restriction
1	Restricted in physically strenuous activity but ambulatory and able to carry out work of a light or sedentary nature, e.g., light house work, office work
2	Ambulatory and capable of all self-care but unable to carry out any work activities; up and about more than 50% of waking hours;
3	Capable of only limited selfcare; confined to bed or chair more than 50% of waking hours;
4	Completely disabled; cannot carry on any selfcare; totally confined to bed or chair;
5	Dead

Table S2. Summary of cancer types within the discovery and validation samples.

Tumour Type	Number of Patients (Discovery)	Number of Patients (Validation)
**Adenoid cystic lung**	1	0
**Adrenal**	1	0
**Ampullary**	0	1
**Bladder**	0	1
**Breast**	13	5
**Cervical**	1	3
**Choroidal Melanoma**	1	0
**Colorectal**	10	22
**CUP**	8	3
**Endometrial**	0	1
**Gastroeosophagael junction**	0	1
**Gall bladder**	0	1
**Lung (NET)**	0	1
**Neuroendocrine**	0	1
**Intrahepatic Cholangio carcinoma**	0	0
**Melanoma**	1	3
**Mesothelioma**	1	0
**NSCLC**	7	9
**Oesophagael**	0	2
**Ovarian**	0	6
**Pancreatic Neuro Endocrine**	1	1
**Penile**	0	1
**Prostate**	1	4
**Rectal**	0	1
**Renal Cell**	1	2
**Sarcoma**	3	1
**Small Bowel**	2	2
**SCLC**	0	4
**Thyroid**	1	0
**Transitional Cell (renal)**	1	0
**Vaginal**	0	1

Table S3. Patient demographics and clinical characteristics .

	Discovery (*n* = 55)	Validation (*n* = 77)		Discovery	Validation
**Sex**			**Smoking status**		
**Female**	35	33	No or former	52	70
**Male**	20	44	Yes	3	6
			Missing	0	1
**Age at consent (years)**			**Number of previous lines of treatment**		
**Median [Range]**	56 [21-82]		Median [Range]	2 [0-12]	2 [0-7]
**<50**	21	15	≤ 1 line	15	26
**≥50**	34	62	> 1 line	40	51
**ECOG PS**			**Number of sites of disease**		
**0**	18	29	Median [Range]	3 [1-6]	2 [1-8]
**1**	35	48	≤ 1 site	8	12
**2**	2	0	> 1 site	47	65
**Baseline albumin**					
**<35 g/L**	1	3			
**≥35 g/L**	51	72			
**Missing**	0	2			
**Baseline LDH (IU/L)**					
**< ULN**	32	50			
**≥ULN**	21	24			
**Missing**	0	3			
**Baseline Hb (IU/L)**					
**< LLN**	2	4			
**≥LLN**	50	71			

Table S4. Summary of AUC values from ROC analysis for Wellness score as a predictor of death in discovery set.

Time period from patients consent to TARGET	AUC	*p* value
**Six months**	0.756	0.002
**Nine months**	0.753	0.003
**12 months**	0.727	0.006
**18 months**	0.709	0.020
**24 months**	0.727	0.021
**36 months**	0.640	0.306

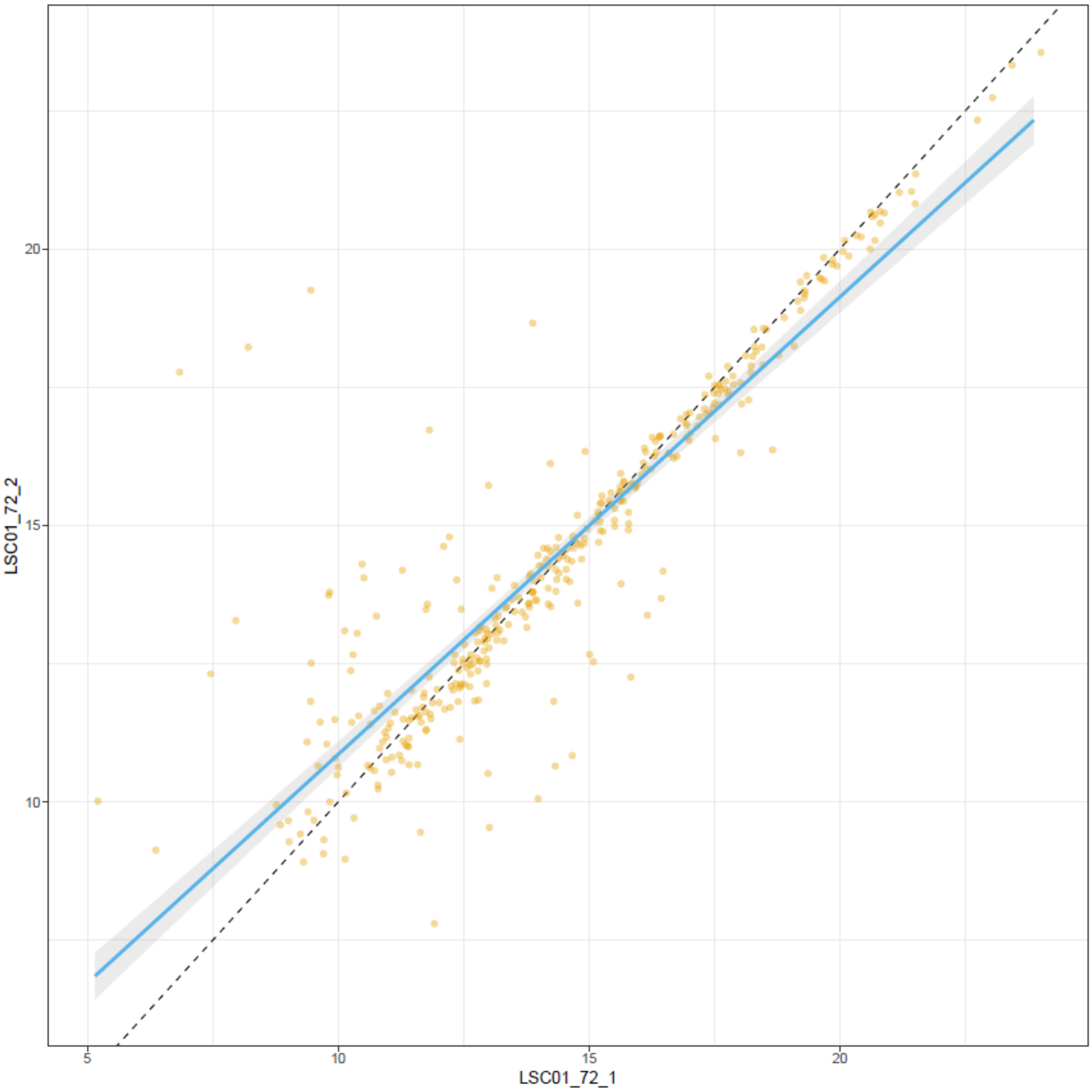

Figure S1. Exemplar correlation plot between two technical replicates. The $\log_2$ protein abundances from two technical replicates from the same biological sample, LSC01_72, are shown. Protein abundances are measured in normalised counts per second. A black dashed line shows a slope of 1. Each orange point represents a unique protein. The light blue solid line shows the linear regression between the two replicates with an adjusted r^2 of 0.8 with a p value of 2.2×10^{-16}.

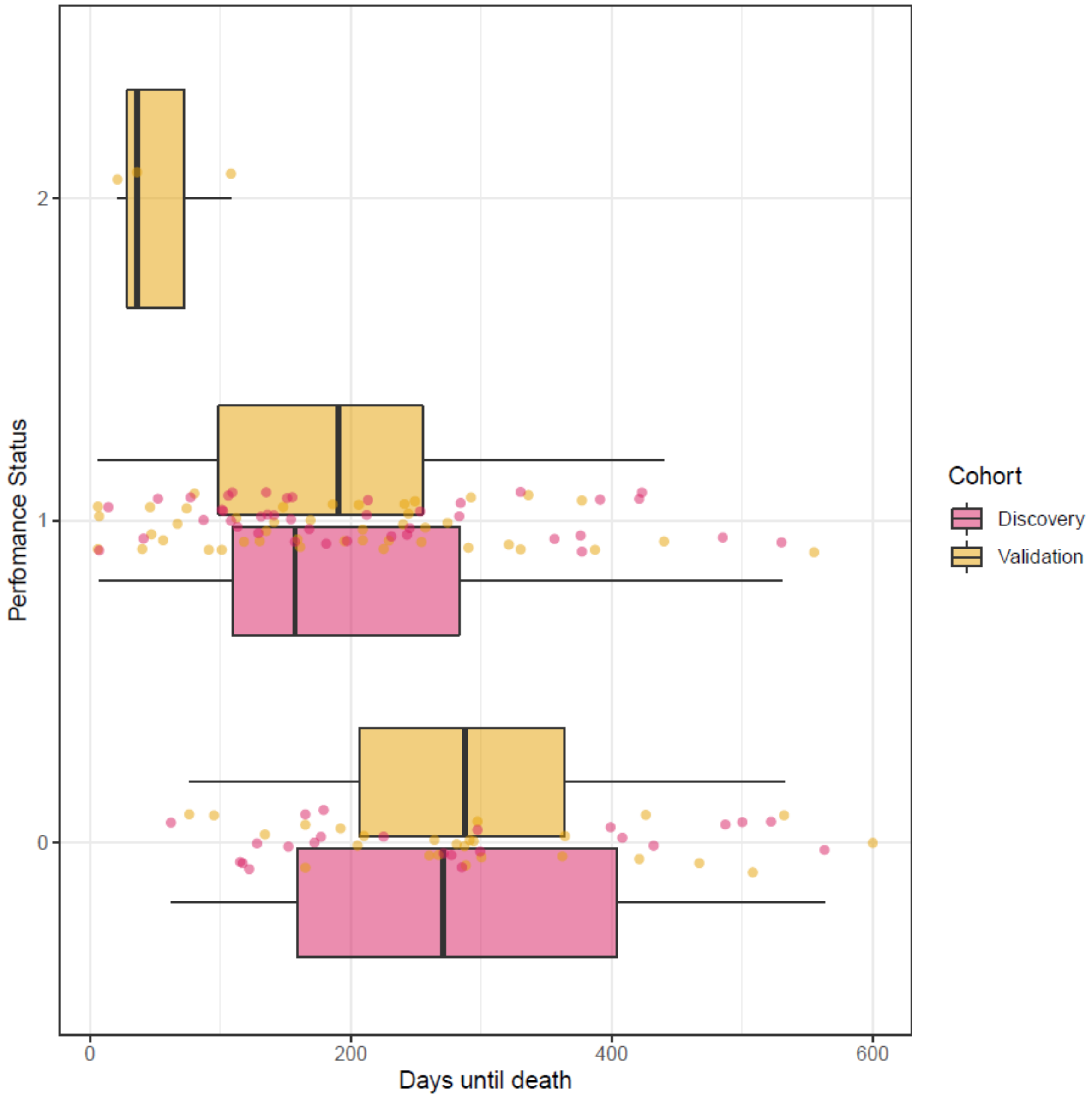

Figure S2. Performance status and time until death in the two cohorts. Each patient is represented as a point with box plots for each cohort. The discovery cohort is coloured in magenta and the validation cohort is shown in gold.

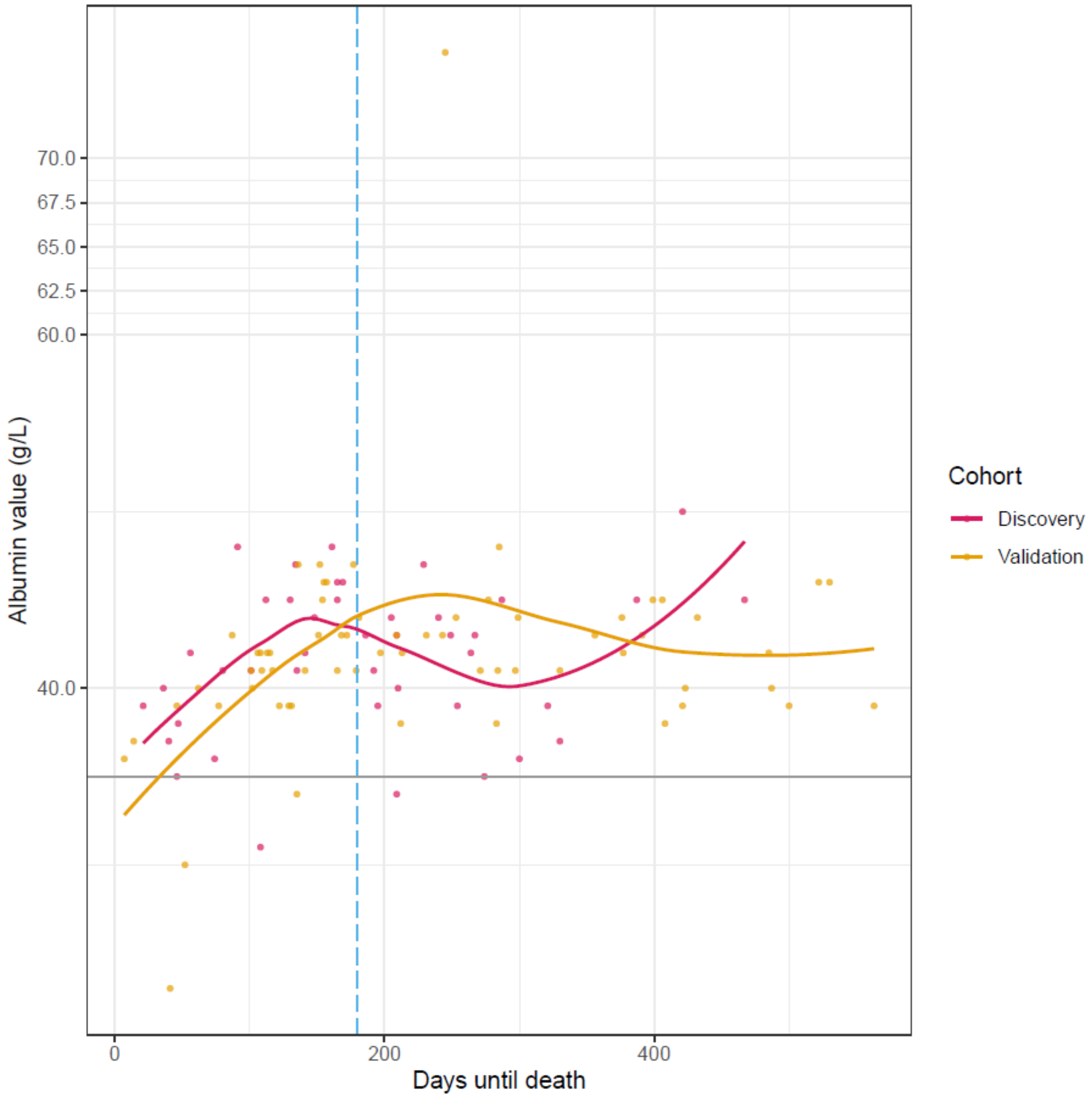

Figure S3. Albumin levels compared against the days until patient death. Each patient was plotted with their serum albumin levels in g/L against the time until death in days. The discovery cohort is coloured in magenta and the validation cohort is shown in gold. Loess curves are plotted for each cohort. A dashed blue line marks six months until death the desired survival length for early phase clinical trials. An albumin value of below 35 g/L, shown by a solid grey line, is considered prognostically poor.

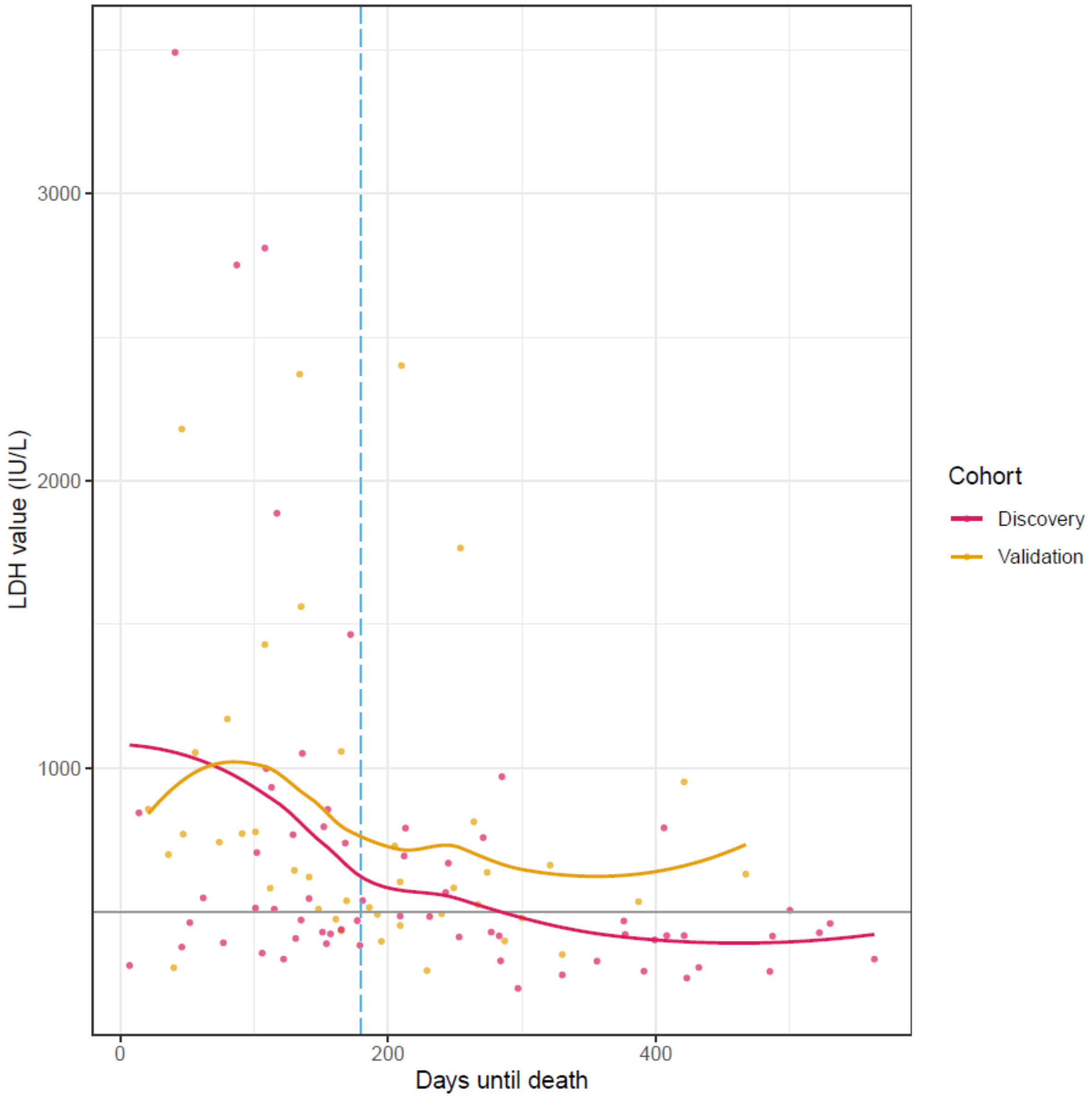

Figure 4. LDH levels compared against the days until patient death. Each patient was plotted with their LDH levels in IU/L against the time until death in days. The discovery cohort is coloured in magenta and the validation cohort is shown in gold. Loess curves are plotted for each cohort. A dashed blue line marks six months until death the desired survival length for early phase clinical trials. An LDH value of above 500 IU/L, shown by a solid grey line, is considered prognostically poor.

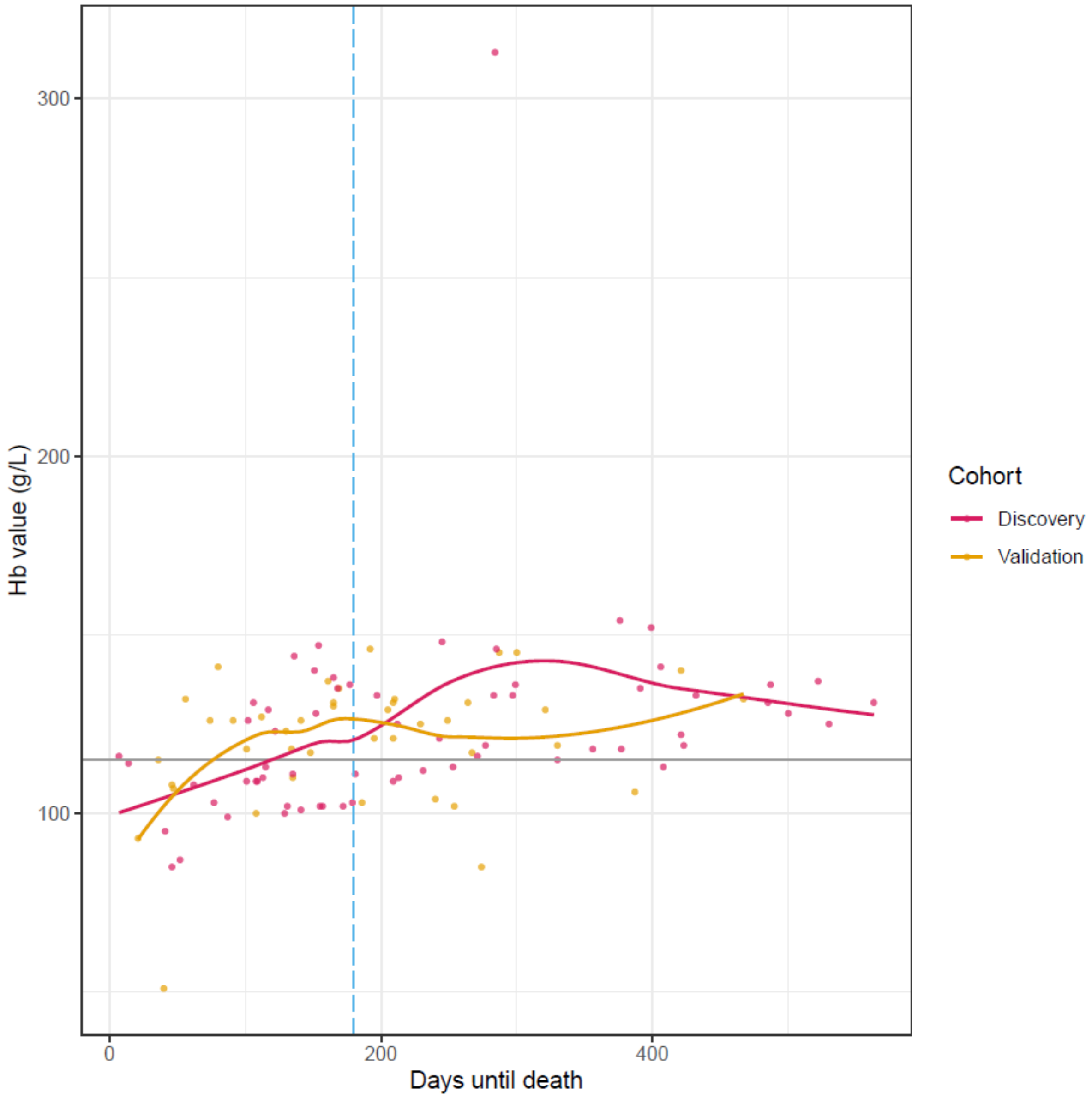

Figure S5. Hb levels compared against the days until patient death. Each patient was plotted with their Hb levels in g/L against the time until death in days. The discovery cohort is coloured in magenta and the validation cohort is shown in gold. Loess curves are plotted for each cohort. A dashed blue line marks six months until death the desired survival length for early phase clinical trials. A Hb value of below 115 g/L, shown by a solid grey line, is considered prognostically poor.

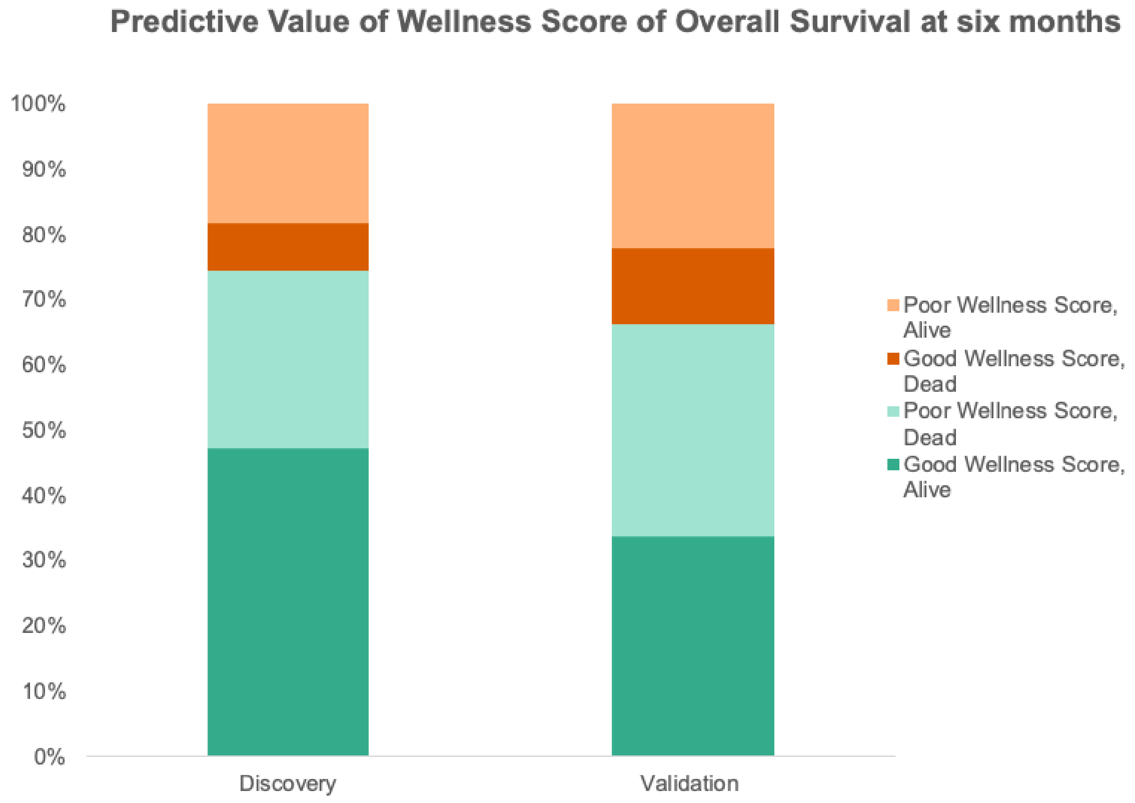

Figure S6. Stacked bar charts showing the proportion of patients stratified by Wellness score and survival status at 6 months in the discovery cohort and the validation cohort. Those that had a good outcome Wellness score and were alive are shown as the dark turquoise segments comprising 48% of total patients in the discovery set and 34% in the validation set. Those that had a good outcome Wellness score and are dead are shown as the light turquoise segments comprising 7% in the discovery set and 12% in the validation set. Patients with a poor outcome Wellness score and were alive at six months are shown as the light orange segments with 18% of all patients in the discovery set and 22% of the patients in the validation set. Patients with a poor outcome Wellness score and were dead within six months are shown in the dark orange segments and made up 27% of the patients in the discovery set and 32% of the validation set.

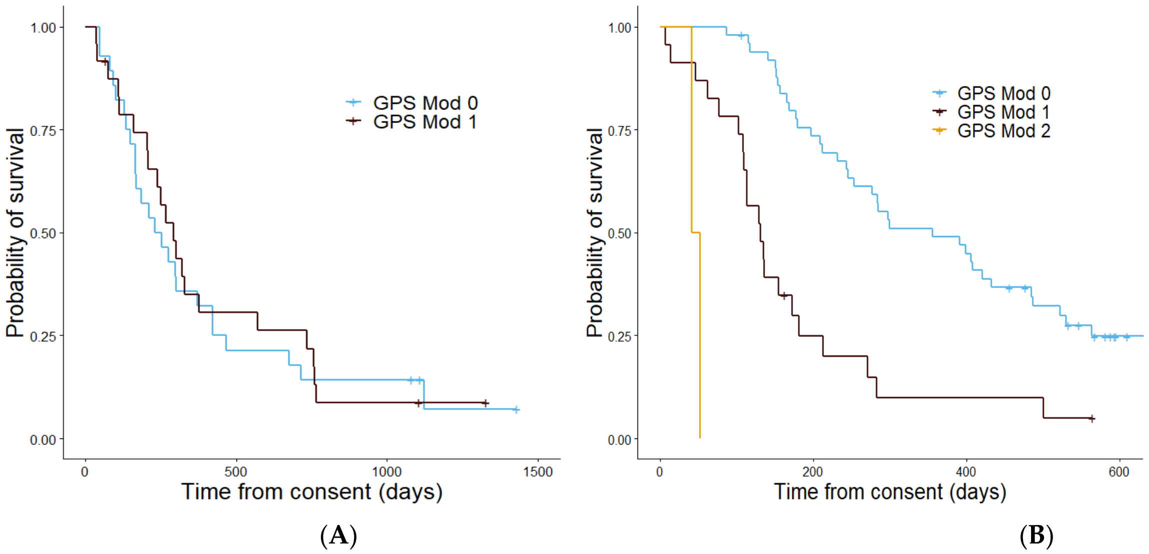

Figure S7. Kaplan-Meier curves showing the overall survival determined by modified GPS in the discovery cohort (A) and the validation cohort (B). In the discovery cohort (A) patients with a modified GPS of 0 had a median estimated overall survival in days of 242, patients with a modified GPS of 1 had an estimated median overall survival of

291 days. The *p* value was 0.774. In the validation cohort (B) the median overall survival estimate in days patients with a modified GPS of 0 was 356, for 1 was 131, and for 2 was 46.5. The *p* value was 2.36 x10 $^{-7}$.

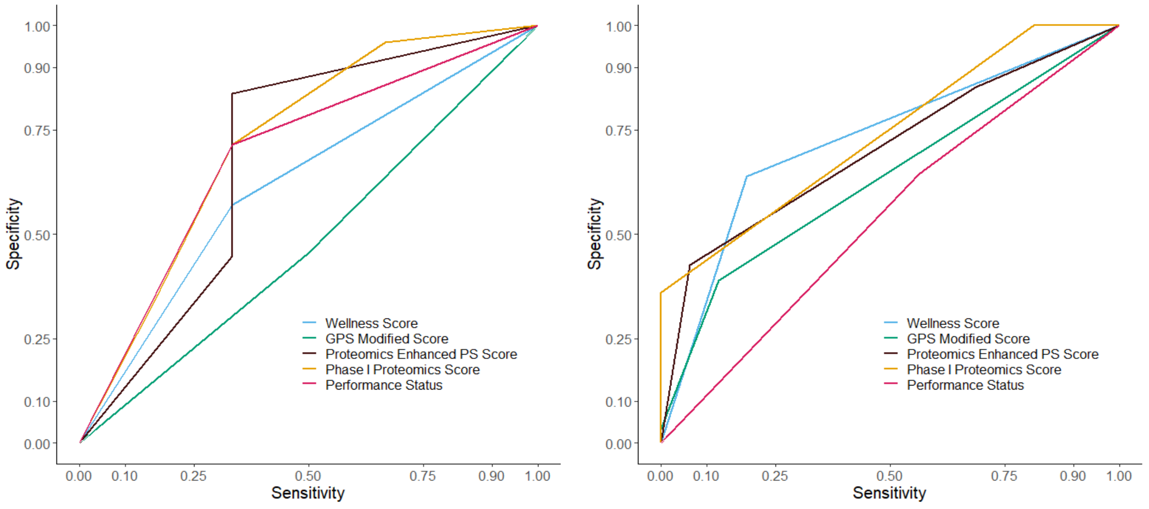

(**A**) (**B**)

Figure S8. ROC curves comparing the different prognostic scores along with modified GPS in the discovery cohort (A) and the validation cohort (B). ROC curves are shown comparing the different scoring metrics in addition to the modified GPS. The AUC of modified GPS in the discovery cohort (A) was 0.478 and 0.634 in the validation cohort (B). AUC values for the other scores are detailed previously in Figure 8.

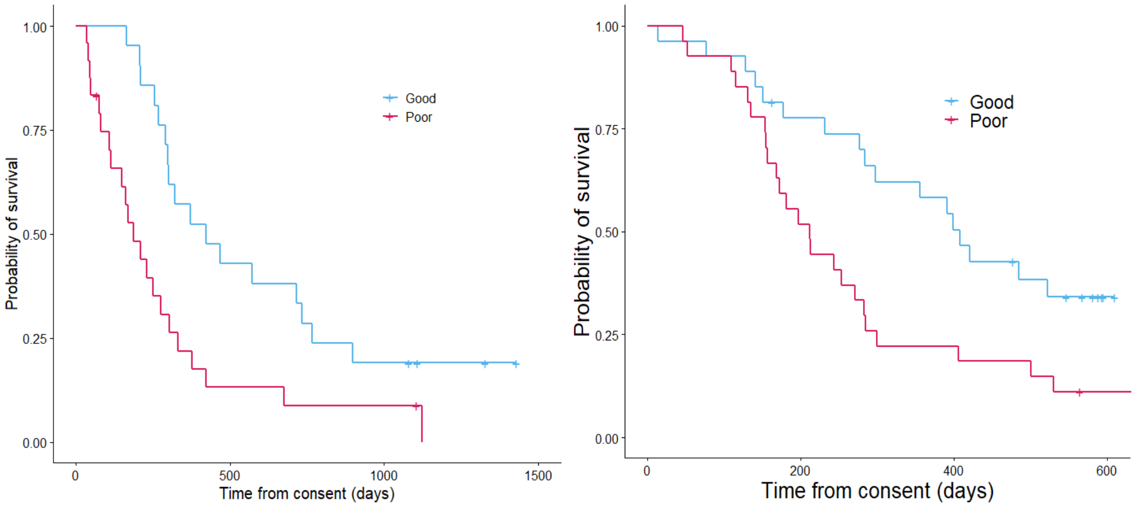

(A) (**B**)

Figure S9. Kaplan-Meier curves showing the overall survival determined by wellness score with colorectal patients excluded in the discovery cohort (A) and the validation cohort (B). In the discovery cohort (A) patients with a good outcome wellness score had a median estimated overall survival in days of 422, patients with a poor outcome wellness score had an estimated median overall survival of 186 days. The *p* value was 0.005. In the validation cohort (B) the median overall survival estimate in days patients with a good outcome wellness score was 408 and for a poor outcome wellness score was 212. The *p* value was 0.0144.
